# Claudin-4: A New Molecular Target for Epithelial Cancer Therapy

**DOI:** 10.3390/ijms24065494

**Published:** 2023-03-13

**Authors:** Rina Fujiwara-Tani, Shiori Mori, Ruiko Ogata, Rika Sasaki, Ayaka Ikemoto, Shingo Kishi, Masuo Kondoh, Hiroki Kuniyasu

**Affiliations:** 1 Department of Molecular Pathology, Nara Medical University, Kashihara 634-8521, Japan; m.0310.s.h5@gmail.com (S.M.); pkuma.og824@gmail.com (R.O.); rika0st1113v726296v@icloud.com (R.S.); dc117023@naramed-u.ac.jp (A.I.); nmu6429@yahoo.co.jp (S.K.); 2 Drug Innovation Center, Graduate School of Pharmaceutical Sciences, Osaka University, 6-1 Yamadaoka, Suita 565-0871, Japan; claudindds@gmail.com

**Keywords:** claudin-4, cancer, molecular target, tight junction, non-tight junction claudin

## Abstract

Claudin-4 (CLDN4) is a key component of tight junctions (TJs) in epithelial cells. CLDN4 is overexpressed in many epithelial malignancies and correlates with cancer progression. Changes in CLDN4 expression have been associated with epigenetic factors (such as hypomethylation of promoter DNA), inflammation associated with infection and cytokines, and growth factor signaling. CLDN4 helps to maintain the tumor microenvironment by forming TJs and acts as a barrier to the entry of anticancer drugs into tumors. Decreased expression of CLDN4 is a potential marker of epithelial-mesenchymal transition (EMT), and decreased epithelial differentiation due to reduced CLDN4 activity is involved in EMT induction. Non-TJ CLDN4 also activates integrin beta 1 and YAP to promote proliferation, EMT, and stemness. These roles in cancer have led to investigations of molecular therapies targeting CLDN4 using anti-CLDN4 extracellular domain antibodies, gene knockdown, clostridium perfringens enterotoxin (CPE), and C-terminus domain of CPE (C-CPE), which have demonstrated the experimental efficacy of this approach. CLDN4 is strongly involved in promoting malignant phenotypes in many epithelial cancers and is regarded as a promising molecular therapeutic target.

## 1. Introduction

Tight junctions (TJs) are multiprotein complexes present at the tip of the lateral membrane of polarized epithelial and endothelial cells [1]. The main function of these structures is to mediate adhesion and polarity between cells. Therefore, it is believed that the impairment of TJs and the concomitant loss of cell-to-cell adhesion are necessary for the early stages of cancer invasion and metastasis. However, it is becoming increasingly clear that TJ proteins such as claudins play important roles not only in adhesion but also in the activation of intracellular signaling, which contributes to tumor progression and metastasis. Claudin (CLDN) is a cell-to-cell adhesion component of the tight junctions and forms a protein family with 27 highly homologous members [2,3]. CLDN4 is the major CLDN involved in TJs in epithelial cells, such as those in the intestines and lungs, and is associated with many epithelial malignancies [4,5]. This review provides an overview of CLDN4 expression, function, and the therapeutic targeting of this protein. In particular, the latest findings are presented on the function of non-TJ CLDN4 and antibody therapy targeting CLDN4 including our data.

## 2. CLDN4 Expression and Regulation in Cancer

Overexpression of CLDN4 has been reported in various cancers, such as gastric cancer [6,7,8], pancreatic cancer [9,10,11], colorectal cancer [12,13], breast cancer [14,15] (especially triple-negative breast cancer [16,17]), oral squamous cell carcinoma [18], ovarian cancer [19], bladder cancer [20,21], non-small cell lung cancer [22], and cholangiocarcinoma [23]. In all of these cases, CLDN4 expression correlated with disease progression and poor prognosis. CLDN4 is also overexpressed in thyroid cancer [24] and prostate cancer [25,26]; however, in these cancers, decreased expression correlated with poor prognosis. Interestingly, in kidney cancer [27] and hepatocellular carcinoma [28], TJ CLDN4 is expressed at low levels and does not correlate with disease progression. 

Several factors have been reported to regulate CLDN4 expression, including epigenetic and inflammatory processes.

### 2.1. Epigenetics

Epigenetic alterations play a major role in carcinogenesis and cancer progression in various malignancies [29,30,31]. Changes in DNA methylation, histone modifications, chromatin remodeling, and microRNAs are considered useful indicators of cancer development and progression [29], and epigenetic changes in the regulation of CLDN4 expression have recently been reported. Hypermethylation of CpG islands in the *CLDN4* promoter region reduces CLDN4 expression in gastric, bladder, and colon cancers [32,33,34]. In contrast, *CLDN4* hypomethylation and CLDN4 overexpression have been reported in gastric, breast, ovarian, and bladder cancers [21,35,36,37]. In bladder cancer, *CLDN4* promoter DNA hypomethylation was shown to correlate with CLDN4 overexpression, high-grade tumors, and invasion, and the increase in CLDN4 promoted anti-apoptosis, stemness, and epithelial-mesenchymal transition (EMT) [21]. Several studies have suggested that the depletion of methyl donors by the upregulation of methyltransferases may be a likely cause of hypomethylation [38,39,40].

Epigenetic regulation of CLDN expression has also been reported for CLDN1 [34,41,42], CLDN2 [43], CLDN3 [32,44,45], CLDN6 and CLDN9 [46], CLDN7 [47], and CLDN11 [48]. Several studies have also indicated the involvement of microRNAs in the regulation of CLDN4 expression. *CLDN4* is a target gene of miR497-3p and the long non-coding RNA ELFN1-AS1, which promotes *CLDN4* expression by sponging miR497-3p [49]. In contrast, coxsackievirus infection suppresses CLDN4 expression and increases airway mucosal permeability by inducing miR4916 expression [50].

### 2.2. Inflammatory Processes

In gastric cancer, CLDN4 expression is elevated in *Helicobacter pylori*-positive cases [8]. Here, CLDN4 expression is upregulated by CDX2, leading to an intestinal phenotype induced by *H. pylori* infection [51]. CLDN4 is downregulated by inflammatory cytokines such as TNFα and HMGB1 [13,52]. In rheumatoid arthritis, blood IL-4, -5, -6, and -13 levels are elevated, while the levels of CLDN4, 7, 12, and 15, as well as ZO-1, are decreased [53]. IL-18 represses the expression of CLDN1, 3, 4, and 12 [54]. Furthermore, endotoxins (in the form of LPS) reduce CLDN4 expression through IL-1β, -6, and -18 [55,56]. In contrast, increased oxidative stress mediated by ROS results in inflammation that upregulates CLDN4 expression through NFκB suppression [57,58]. Increased expression of CLDN4 in acute pancreatitis is reportedly due to enhanced transcription by FOXP3 and USF2 [59]. These findings suggest that changes in CLDN4 expression may result from the balance between inflammation, cytokine activity, and inflammation-associated ROS production.

### 2.3. Growth Factors

Smad signaling triggered by TGF-β induces CLDN4 promoter activity via c-Jun, enhancing CLDN4 expression [60]. In mouse intestinal epithelium, knockdown of smad4 has been shown to reduce the expression of CLDN3 and 4, but increase that of CLDN2 and 8, resulting in increased intestinal permeability [61]. In glioblastoma, TGFβ promotes CLDN4 expression and enhances invasive ability [62]. Other signaling pathways that affect CLDN4 expression include PKCα [63], twist [64], ERK1/2 [65], p38MAPK [66], HIF1α [67], and hedgehog [68].

## 3. The Function of CLDN4 in Cancer

### 3.1. Carcinogenesis

CLDN4 overexpression has been detected in several cancers, including lung, gastric, colorectal, endometrial, uterine cervical, and ovarian epithelial cancers. In these cancers, precancerous lesions, atypical adenomatous hyperplasia, gastric dysplasia, sessile serrated adenoma/polyp with dysplasia (SSA/P-D), atypical endometrial hyperplasia, cervical intraepithelial neoplasia (CIN), and borderline malignant lesions display increased expression and/or abnormal distribution of CLDN4 [69,70,71,72,73,74]. Furthermore, even in liver cancer with a low level of CLDN4 expression, CLDN4 expression is increased in liver cirrhosis compared to normal liver tissue [75]. Thus, CLDN4 overexpression appears to represent an early event in carcinogenesis in many cancers, and YAP activation by CLDN4 has been reported as a molecular biological background in precancerous lesions of colorectal cancer [71]. In papillary thyroid carcinoma, an oncogenic chromosomal gain of 7q11.22-11.23 leads to CLDN4 upregulation [76]. In contrast, there are also reports suggesting that the expression of CLDN4 suppresses carcinogenesis. CLDN4 knockout mice exhibit a hyperproliferation of the urothelium [77], while activation of the tumor suppressor gene cancer-related regulator of actin dynamics (CRAD) is associated with CLDN4 upregulation [78]. CLDN4 inhibits EphA2 oncogenic signaling by inactivating the β-catenin and PI3K-AKT pathways [79].

### 3.2. Barrier Function and Maintenance of Intratumoral Microenvironment

**Figure 1:**
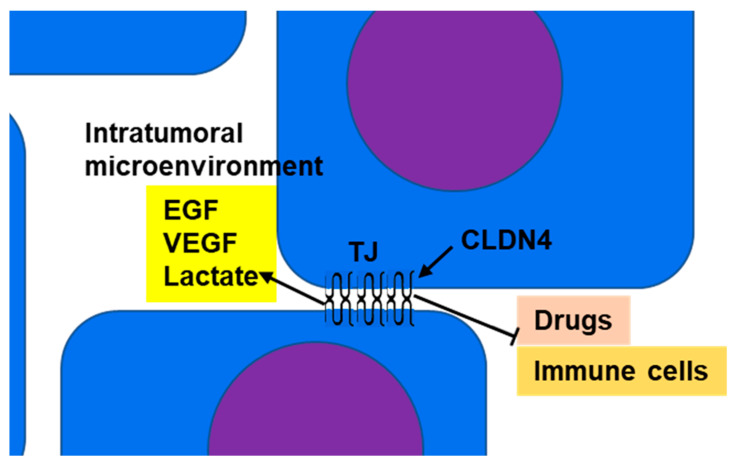
Barrier function of TJs: CLDN4, which is overexpressed in epithelial malignancies, separates the intratumoral microenvironment from the tumor exterior by forming TJs. As a result, growth factors (such as EGF and VEGF) and metabolites (such as lactate) accumulate in the intratumoral microenvironment, resulting in amplification of their effects. This promotes increased tumor grade and suppression of immune cell infiltration into the tumor. TJs also inhibit the penetration of anticancer drugs from the tumor exterior into the microenvironment and enhance resistance to anticancer drugs. CLDN4, claudin-4; TJ, tight junction; EGF, epidermal growth factor; VEGF, vascular endothelial growth factor.

CLDN4 is a major structural protein of epithelial TJs in intestines and lungs and is involved in epithelial differentiation, polarity maintenance, and substance trafficking [80,81]. In normal epithelial tissue, TJs act as barriers or gates that separate the outside from the inside of the body and restrict material transport; however, in tumor tissue, the polarity of the cells and tissues is ambiguous. Thus, in CLDN4-overexpressing epithelial malignancies, the barrier function of TJs serves to maintain the tumor microenvironment and retain tumor-secreted growth factors to promote the malignant cancer phenotype (Figure 1) [8,11,13,16,20]. VEGF and IL-8, cancer cell-derived angiogenic factors, are upregulated by CLDN4 and their accumulation in tumors promotes tumor angiogenesis [20,82]. In addition, cancer cells exhibit enhanced glycolysis (known as the Warburg effect) that results in the release of extracellular lactate [83]. The barrier function of CLDN4 leads to the accumulation of lactate within the tumor microenvironment and maintains an acidic environment around the cancer cells [16]. A decrease in extracellular pH is associated with the pH-sensitive G-protein-coupled receptors (GPCRs) GPR4, GPR65 (TDAG8), GPR68 (OGR1), and GPR132 (G2A). This acidic environment promotes the proliferation, migration, invasion, and metastasis of cancer cells. An acidic tumor microenvironment, linked with a hypoxic environment, promotes the expression of malic enzyme 1 and brings EMT to tumor cells through YAP1 activation [83]. An acidic environment also suppresses immune cells (allowing cancer cells to escape from cancer-targeted immunity) [84]. Furthermore, TJs restrict the movement of external substances into the tumor microenvironment, thus enabling cancer cell drug resistance [85]. 

In assessing the barrier action of TJ CLDN4, it is necessary to consider its involvement in transport in normal TJs. TJ controls material transport through the paracellular and transcellular routes. Utilization of these pathways is affected by the ratio of ionized and unionized species (which depends on the pKa of the drug, the size of the molecules, and the pH of the solution), the intrinsic partition coefficient of the drug, and the size of the molecule and its charge [86]. For example, most of the filtered Cl- is reabsorbed in the proximal tubules. A key component of Cl reabsorption is passive, paracellular, driven by the luminal-negative potential of the early proximal tubule and the outward concentration gradient for Cl in the late proximal tubule. Moreover, CLDN4 forms paracellular chloride channels in the kidney, and CLDN8 is required for tight junction localization [87].

Small intercellular distances, high rates of drug influx into cells, low rates of drug efflux, and high intracellular and extracellular drug binding promote the development of drug gradients. In the absence of drug metabolism, the gradient “levels out” over time and may even reverse when blood concentrations decrease. Understanding the drug transport process from microvessels to individual cancer cells is important for optimizing cancer chemotherapy. Cancer cells that can ‘hide’ from drugs may lead to tumor regrowth [88].

### 3.3. Apoptosis

Several studies have suggested that CLDN4 expression is involved in promoting cancer cell viability. CLDN4 suppresses cell death caused by apoptosis [15,89] and endoplasmic reticulum stress [90]. It is believed that non-TJ CLDN4 activates integrin β1 as a binding partner and suppresses apoptosis via FAK signaling [8,21]. In addition, CLDN1 [91,92] increases resistance to anoikis, whereas CLDN6 decreases it [93]. However, the effect of CLDN4 on anoikis is still unclear.

### 3.4. Stemness and EMT

In EMT, epithelial cells lose epithelial differentiation and transition to mesenchyme, which involves background dedifferentiation from the epithelium and enhanced stemness [94]. It has become clear that cancer stemness is the basis of tumorigenicity, self-renewal, and differentiation, as well as tumor heterogeneity, metastasis, and treatment resistance [95]. Decreased expression of CLDN4, an epithelial marker in tumors, reflects EMT and is also associated with metastasis [96,97]. EMT correlates with hypermethylation of the *CLDN4* promoter, which causes downregulation of CLDN4 [98], while the transcription factor Bach1 directly suppresses the expression of *CLDN4* and *E-cadherin* to induce EMT [99]. Thus, CLDN4 expression is a marker of epithelial traits, and its decrease is thought to be suggestive of EMT. However, Ma et al. reported that repression of CLDN4 expression suppresses invasion and metastasis in breast cancer cell lines [15]. Accumulating knowledge indicates that CLDN4 plays a role in maintaining epithelial differentiation. CLDN4 knockdown results in enhanced cell proliferation and invasive capacity, suppression of apoptosis, and promotes metastatic potential [100,101]. While there was no induction of vimentin expression, the authors did observe decreased E-cadherin and increased N-cadherin expression, suggesting EMT. Analyses of the signaling changes underpinning this phenomenon show that decreased CLDN4 expression leads to GSK-3β phosphorylation, Wnt signal activation by β-catenin nuclear translocation, PI3K/AKT signal activation, and the induction of Twist expression [100,101]. This results in enhanced proliferation and anticancer drug resistance [102].

## 4. Non-TJ Functions of CLDN4

Although CLDN4 acts primarily as a structural protein in tTJs, research has revealed that this protein also exhibits a diverse range of non-TJ functions [103]. These include functions of membrane-bound CLDN4 outside of TJs, as well as free cytoplasmic CLDN4.

### 4.1. Non-TJ Plasma Membrane CLDN4

CLDN4 is overexpressed in bladder cancer due to promoter DNA hypomethylation [21]. Further demethylation via aza-2′-deoxycytidine (AZA) treatment induces expression of CLDN4 to levels above that necessary for TJ function. This is accompanied by the formation of CLDN4 monomers that do not incorporate into TJs [21]. This process is considered to be one of the mechanisms responsible for generating non-TJ CLDN4. In gastric cancer, plasma membrane CLDN4 is overexpressed in well-differentiated carcinomas, whereas plasma membrane CLDN4 that forms a TJ with E-cadherin is decreased in poorly differentiated carcinomas, reflecting EMT phenotype. In contrast, non-TJ CLDN4 is increased. Non-TJ CLDN4 is associated with EMT phenotypes [8].

### 4.2. Cytoplasmic CLDN4

CLDN4 can also be taken from TJs to form a non-plasma membrane (cytoplasmic) CLDN4. Studies have demonstrated that the C-terminus domain of *Clostridium perfringens* enterotoxin (CPE) binds to the second extracellular loop of CLDN4, disrupting homotypic claudin binding, impairing TJs, and leading to diarrhea [104]. As a result, CLDN4 is released from TJs and translocated into the cytoplasm [13,18]. In addition, when EphA2 on the plasma membrane is activated by binding to its ligand Ephrin A1, the C-terminal tyrosine residue of CLDN4 in the adjacent TJ is phosphorylated by EphA2, promoting the release of CLDN4 from the TJ and resulting its translocation into the cytoplasm [27]. Furthermore, cytoplasmic CLDN4 is transported into the nucleus after phosphorylation of the C-terminal serine residue by protein kinase C (PKC)-ε [27].

### 4.3. Function of Non-TJ CLDN4

#### 4.3.1. Integrin β1 Activation

**Figure 2:**
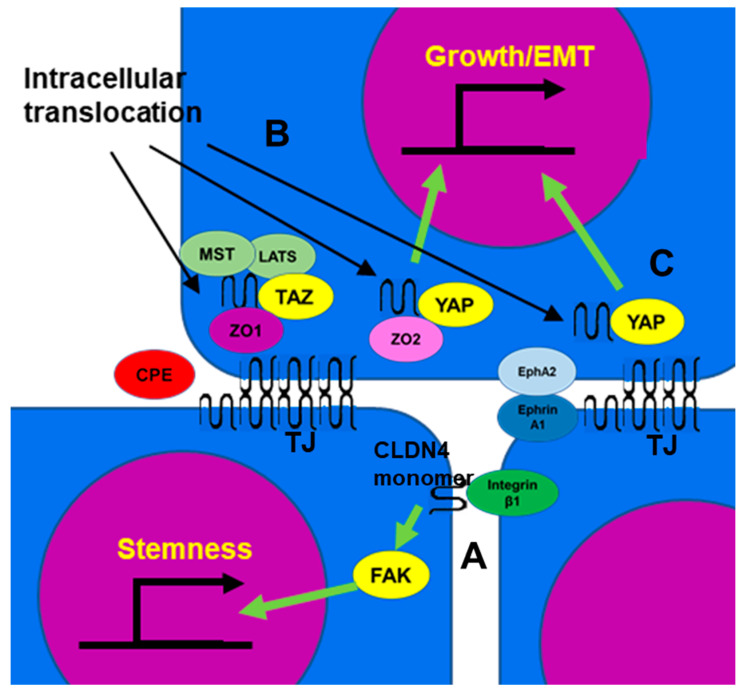
The function of non-TJ CLDN4. (A) CLDN4 monomer that does not form TJs is a binding partner for integrin β1 expressed in neighboring tumor cells, activates FAK, and promotes expression of stemness-associated genes. (B) CLDN4 translocates into the cytoplasm from TJs disrupted by CPE and forms a stable complex with TAZ, MST, LATS, and ZO1, but YAP is released from the complex and translocates into the nucleus with ZO2 to induce expression of YAP target genes, leading to proliferation and EMT. (C) EphA2 activated by Ephrin A1 expressed on the surface of neighboring cells phosphorylates CLDN4 and releases it from TJs. The released CLDN4 is translocated into the nucleus with YAP. CLDN4, claudin-4; TJ, tight junction; CPE, *Clostridium perfringens* enterotoxin; TAZ, tafazzin; YAP, yes-associated protein; ZO, zonula occludens; MST, mammalian Ste20-like kinase; LATS, large tumor suppressor kinase; Eph A2, ephrin type-A receptor 2; FAK, focal adhesion kinase; EMT, epithelial-mesenchymal transition.

Integrin β1 activates FAK and induces the expression of stem cell-related genes such as Oct4, Sox2, and Nanog through Notch signaling [105,106]. Non-TJ CLDN4 binds to integrin β1 and enhances stemness, anti-apoptotic effects, drug resistance, and metastatic capacity of cancer cells (Figure 2A) [8,21]. In poorly differentiated gastric cancer, TJ formation is reduced, but EMT is mediated by non-TJ CLDN4 [8]. CLDN7, like CLDN4, also binds to integrin β1, leading to downstream FAK phosphorylation [107,108]. CLDN4 exhibits approximately 40% of the affinity of CLDN7 for integrin β1 [8].

#### 4.3.2. YAP Activation

Cytoplasmic CLDN4 is also involved in YAP activation [18,27,71]. CLDN4 translocated to the cytoplasm by *C. perfringens* CPE in the intestinal flora forms a stable complex involving TAZ, LATS, MST of HIPPO inhibitory system, and ZO-1. This sequestration of the YAP co-activator TAZ leaves YAP free to bind to ZO-2 and translocate to the nucleus, where it promotes the expression of target genes such as *cyclin E* and *snail*, stimulating proliferation and inducing EMT (Figure 2B) [71]. As a result, it promotes carcinogenesis of SSA/P-D, a colonic precancerous lesion, and is associated with BRAF mutations in colorectal cancer [71]. In contrast, in oral squamous cell carcinoma, CLDN4 released into the cytoplasm by CPE from *C. perfringens* in the oral flora binds to YAP and ZO-2 and translocates into the nucleus, promoting proliferation and inducing EMT. [18]. Nuclear CLDN4 is found in 39% of oral squamous cell carcinomas and 81% of oral *C. perfringens*-positive cases [18]. In renal cell carcinoma, unlike many epithelial malignancies, CLDN4 expression level is low, but nuclear CLDN4 is observed in 2% of tumors, all of them at an advanced stage [27]. As mentioned earlier, in renal cell carcinoma, EphA2/Ephrin A1 and PKCε translocate CLDN4 into the nucleus, with YAP bound and co-translocated alongside CLDN4. As a result, YAP is activated and increases the malignancy of cancer cells (Figure 2C) [27]. YAP activation by CLDN also occurs with CLDN6 and CLDN18. Several factors are involved in YAP nuclear translocation and activation, including the inhibition of YAP phosphorylation by LATS [109], CLDN6-ZO2-YAP interactions [110], and the binding of CLDN18 with YAP [111], which together lead to a poor prognosis in gastric cancer [112].

#### 4.3.3. Activation of AKT

CLDN4 has also been linked to AKT signaling. CLDN4 has been shown to induce PIK3R3 and MAP2K2 mRNA expression and activate AKT and ERK1/2 in acute myeloid leukemia cells [113]. This results in accelerated proliferation and poor prognosis for this disease. Another study indicated that SPTBN2 cooperates with CLDN4 to stimulate PI3K/AKT activation [114]. Conversely, there is also a report that silencing CLDN4 activates AKT [102]. CLDN4 limits the activity of β-catenin and PI3K and inhibits the phosphorylation and activity of EphA2 by AKT [79].

## 5. Targeting CLDN4

The overexpression of CLDN4 in many cancers has drawn attention to this protein as a new molecular target. There have been a number of attempts to target CLDN4 for cancer therapy. Targeting of CLDN4 is expected to provide multi-layered effects by enabling direct attacks on CLDN4-overexpressing cancer cells, disrupting the intratumoral microenvironment, and facilitating drug delivery by impairing TJs. It is also expected to inhibit tumor-promoting signals generated by non-TJ CLDN4.

### 5.1. Antibodies

To target CLDN4 with antibodies, it is essential to generate an antibody against its extracellular domain, but it is difficult to generate a single CLDN-specific antibody due to the high homology among CLDN family members [115]. The antibodies reported to be established thus far include monoclonal antibodies that recognize the extracellular loops of both CLDN3 and CLDN4 and their antitumor effects have been confirmed both in vitro and in vivo [116,117]. Suzuki et al. generated a monoclonal antibody (KM3900) that recognizes CLDN4 extracellular loop 2 and induces antibody-dependent cytotoxicity (ADCC) and complement-dependent cytotoxicity (CDC) in vitro and inhibited the growth of pancreatic and ovarian tumors in SCID mice in vivo [118]. Using DNA immunization, Kuwada et al. produced a monoclonal antibody (4D3) that recognizes CLDN4 extracellular loops 1 and 2 and confirmed its antitumor effect in a nude mouse model [20]. The 4D3 antibody induces ADCC and CDC, but its particular advantage is its sensitizing effect on anticancer drugs by promoting the delivery of anticancer drugs into the tumor microenvironment due to TJ damage [20]. As a result, the antitumor effect of combining 4D3 with anticancer agents such as CDDP, 5-FU, paclitaxel, and folfirinox has been observed in animal models of bladder, colon, gastric, pancreatic, and breast cancers [8,11,13,16,20]. In addition, the 4D3 antibody also has sensitizing effects on cetuximab, tamoxifen, and bisphosphonates [13,16]. In addition, disruption of TJs by 4D3 reduces high levels of stored growth factors and lactates within the tumor microenvironment, promoting antitumor effects [16,20].

Since CLDN4 is expressed in various epithelial tissues, it is essential to ensure that antibodies targeting CLDN4 are safe for human use [2]. In a study using mouse anti-CLDN4 antibody, the anti-CLDN4 antibody did not show any marked alteration in the distribution in the body (compared to non-specific IgG) in tumor-bearing mice and demonstrated higher accumulations in the tumors of these animals. Furthermore, no specific organ damage was observed [119].

Although there are, as yet, no clinical trials underway testing anti-CLDN4 antibodies, clinical trials of antibody drugs against CLDN6 and CLDN18 have begun [120,121], and testing for clinical application of anti-CLDN4 antibodies is expected.

### 5.2. Knockdown

Knocking down CLDN4 in gastric cancer and bladder cancer results in a mild decrease in transepithelial electrical resistance (TER), an indicator of TJ function [8,21]. For this reason, CLDN4 knockdown only provides limited disruption of the microenvironment and the promotion of anticancer drug permeability by impairing TJs. One possible reason for this is that the knockdown of a single CLDN may result in other CLDNs maintaining TJ function in its place. However, CLDN4 knockdown does reduce non-TJ CLDN4 and thus inhibits stemness [21].

### 5.3. CPE and C-Terminus Domain of CPE (C-CPE)

CPE recognizes specific amino acid sequences in the first and second extracellular loops of CLDN4 and CLDN3 and docks via a pocket of the domain at the C-terminus to disrupt TJs. Furthermore, it perforates the plasma membrane to cause cell death due to the intracellular influx of calcium [104,122]. Therefore, CPE exhibits cytotoxicity against cancer cells expressing CLDN4. The antitumor effect of CPE has been demonstrated by experiments in prostate cancer [123,124], non-small cell lung cancer [22], pancreatic cancer [10], gastric cancer [125], and ovarian cancer [126,127].

C-CPE is a C-terminal fragment of CPE. Like CPE, it binds to CLDN4 and CLDN3 and impairs TJs; however, unlike CPE, it does not perforate the cell membrane [128]. Impairment of TJs by C-CPE disrupts the barrier of the tumor microenvironment and facilitates drug delivery [129], which enhances anticancer drug susceptibility [130] and suppression of metastasis [131]. Furthermore, by conjugating toxins and anticancer drugs to C-CPE, it becomes a carrier that delivers these to cancer cells expressing CLDN4 [63]. For example, *Pseudomonas aeruginosa* exotoxin A [132,133], diphtheria toxin fragment A [134], doxorubicin-loaded polysialic acid nanoparticles [135], ^111^In [136], TNF [137], and nanomaterials such as gold nanoparticles [138] bound to C-CPE induces cell death in CLDN4-expressing cancer cells. However, the immunogenicity and potential toxicity of CPE may limit its clinical application [139]. Just as C. perfringens causes food poisoning, CPE damages the mucosal epithelium and marked cytokine reaction, resulting in gastrointestinal disorders and CPE-induced shock [140,141]. In addition, C-CPE may bring about cytoplasmic translocation of CLDN4 in the same way as CPE, and it is necessary to analyze the effects on YAP activation and other CLDN interacting partners (see the section on YAP activation).

### 5.4. Peptide

Attempts have also been made to produce specific peptides as CLDN binding agents. Hicks et al. showed that a small peptide that mimics the DFYNP sequence in the second extracellular loop of CLDN4 impairs CLDN4, leading to the induction of apoptosis and suppression of tumor growth [89]. In light of these promising data, further progress is expected in peptide drug discovery to target CLDN4.

### 5.5. Delivery of Anti-CLDN4 Drugs

In many cases, CLDN4-targeting drugs such as those described above reach the tumor through blood administration. At this time, the formation of tumor blood vessels is important for the effective delivery of molecular-targeted drugs. As mentioned above, the barrier action of CLDN4 leads to the accumulation of angiogenic factors within the tumor microenvironment and may promote angiogenesis. However, in the future, a more detailed examination is required regarding tumor blood vessels and the delivery of molecularly targeted drugs. Drugs that use the extracellular route are more hydrophobic in nature, whereas drugs that can pass through intercellular spaces are more hydrophilic. The nature of such agents in CLDN4 targeting also needs to be considered. As mentioned in the antibody section, CLDN4 is expressed in a variety of normal tissues, and off-target effects of anti-CLDN4 drugs other than antibodies should also be investigated. 

## 6. Conclusions

CLDN4 has a well-established role in cell adhesion as a TJ protein. However, recent studies have revealed that CLDN4 is not only involved in cell adhesion but also in signal transduction, which plays an important role in the formation of cancer pathologies such as tumor initiation, progression, and metastasis. Our expanding knowledge of the functions of TJs and elucidation of the role of non-TJ CLDN4 has revealed the variety of CLDN4 functions and increased awareness of its importance in cancer. Various molecular therapies that target CLDN4 are also being developed and are expected to result in new therapeutic approaches that exhibit efficient antitumor effects when used in combination with chemotherapy. The difficulty of developing CLDN4-specific molecular-targeted drugs due to the high homology among the claudin family proteins and the fact that the protein is also widely expressed in normal tissues are barriers to CLDN4 targeting. Many other issues, such as the diverse role of CLDN4 in tumors, interactions with other CLDNs, and substance penetration by TJ CLDN4, complicate CLDN4 targeting. Even with these considerations, it is emphasized that targeting CLDN4 is an attractive therapeutic approach that offers pleiotropic benefits, including cancer cytotoxicity, reprogramming tumor microenvironments, and improvement of drug delivery.

## Data Availability

Not applicable.

## Abbreviations

TJ, tight junction; CLDN, claudin; EMT, epithelial-mesenchymal transition; YAP, yes-associated protein; CPE, clostridium perfringens enterotoxin; C-CPE, C-terminus domain of CPE; CDX2, caudal type homeobox2; TNF, tumor necrosis factor; HMGB, high mobility group B; IL, interleukin; LPS, lipopolysaccharide; ROS, reactive oxygen species; NFκB, nuclear factor κB; FOXP3, forkhead box P3; USP2, upstream transcription factor 2; TGF, transforming growth factor; PKC, protein kinase C; MAPK, mitogen-activated protein kinase; ERK, extracellular signal-regulated kinase; HIF, hypoxia-inducible factor; SSA/P-D, sessile serrated adenoma/polyp with dysplasia; CIN, cervical intraepithelial neoplasia; CARD, cancer-related regulator of actin dynamics; PI3K, phosphoinositide 3-kinase; VEGF, vascular endothelial growth factor; GPCR, GPR, G-protein-coupled receptor; TDAG8, T cell death-associated gene 8; OGR1, Ovarian cancer G-protein-coupled receptor 1; G2A, G2 accumulation; FAK, focal adhesion kinase; Bach1, BRCA1-interacting protein C-terminal helicase 1; GSK, glycogen synthase kinase; AZA, aza-2’-deoxycytidine; Oct, octamer-binding transcription factor; Sox, sex-determining region Y-box; LATS, Large tumor suppressor homolog; MST, macrophage-stimulating; TAZ, transcriptional co-activator with PDZ-binding motif; ZO, zona occludens; PIK3R3, phosphoinositide-3-kinase regulatory subunit 3; MAP2K2, MAPK kinase 2; SPTBN2, spectrin beta chain, non-erythrocytic 2; ADCC, antibody-dependent cytotoxicity; CDC, complement-dependent cytotoxicity; CDDP, cis-diamminedichloroplatinum; 5-FU, 5-fluorouracil; TER, transepithelial electrical resistance.
